# The Clinical Features of Bilharzia Hæmatobia

**Published:** 1903-07-11

**Authors:** 


### THE CLINICAL FEATURES OF BILHARZIA HÆMATOBIA.

Cases of bilharzia hæmatobia were at one time of very great rarity in England, but though still somewhat of a medical curiosity, they have been more commonly met with of late years than was formerly the case. The endemic home of the parasite is believed to be chiefly Africa, if not Africa alone, and the opening up of this continent, east, west, north and south, has led to a large number of men of a class rather different to that of the older travellers and explorers being despatched thither in connection with the working of mines and the building of railways.

The life history of the parasite is as yet but ill understood, and although the disease has hitherto only been observed in those who have visited or been resident in its endemic home, it is by no means certain that it is incapable of flourishing in other climates. However this may be, cases of the disease may now be met among another class of men than that of returned residents in Africa.

It is certain that cases of it were fairly common in many of the military hospitals in South Africa during the war, and that large numbers of infected men have since scattered over Great Britain, India, Australia, Canada, and other parts of the world. Of such cases six have recently been under treatment at the Herbert Hospital, Woolwich, and a good many other men have been discharged from the regular army on account of it. Cases therefore are now liable to be met with in the ordinary course of private practice, and, in consequence, a consideration of the clinical features of the disease has a more direct interest to men in general practice than formerly.

It is believed that the parasite, a trematode, passes some portion of its existence in an unidentified fresh water organism. How it gains access thence to the human body is uncertain but once installed it may take up its quarters in any part of the venous system. Post-mortem it is usually found either in the portal vein itself or in one of its radicals or branches, but as this is not necessarily or invariably the case, the symptoms caused by the protrusion of its ova in submucous and other tissues are liable to variation. In nearly all cases, however, recognised ante-mortem, symptoms referable to the bladder and ureters are much more common than any other. In fact, hæmaturia is so commonly present that the disease was known as "Endemic Hæmaturia" long before its precise cause was identified by Bilharz in Egypt in 1851.

There are many good descriptions of its pathology, but perhaps the best and most elaborate is that of the Italian observer, Sonsino.[1] The latter's experience, however, was gained in Egypt, and for the description of the class of case likely to be met with in English practice a reference may be made to a clinical lecture by Dr. J. H. Bryant[2] on some cases under his care.

The symptoms vary considerably, but usually more in degree than in kind. In some cases they are so slight that the patient, unless of a nervous type, experiences very little or no trouble at all. He may simply notice that, with more or less regularity, he passes a drop or two of dark or bloody urine at the end of micturition. From this it may vary to the occasional passage of almost pure blood. In many cases the earliest symptom of ill health has been a sudden attack of diarrhœa, accompanied by the passage of blood and mucus. Not uncommonly the case resembles mild intermittent dysentery, and consequently the urine of such patients should always be specially examined. Micturition may be painful towards the end of the act, when blood clot and *débris* are passed, and, on active exercise, aching very commonly occurs in the loins, perineum, thighs, inguinal or hypogastric region.

If the veins round the prostate and vesiculæ seminales are affected there may be, besides perineal pain, priapism and seminal emissions. When the neck of the bladder is affected there is often vesical tenesmus. In long severe cases the loss of blood and pain give rise to anæmia, lassitude, and fibrillary muscular tremors, and complications such as cystitis, urinary calculi, and occlusion of the ureter are liable to occur. In lower Egypt the occurrence of calculi in connection with Bilharzia is very common. It is to be remembered, however, that very often the symptoms are by no means of a severe and marked character. In such cases the patients look strong and well, and make no complaint beyond that of occasional pain and hæmaturia, and the true condition is liable to be overlooked. In Natal, indeed, where the disease is extremely common among boys, it is regarded almost as a physiological process, and the boys apparently take little notice of it.

The changes in the urine are characteristic. In early cases the urine is clear, but shows on close inspection minute specks of scarlet. Later on, the first part is passed clear, but at the end of micturition a drachm or so of mucus and blood is voided. The amount of urine is normal, and also its reaction, except in cases where cystitis has occurred. If the deposit on standing is examined under the microscope, besides blood and pus cells, the ova, with their characteristic spines, are readily found, and adding a little warm water to the deposit often causes the ova to develop on the slide. In some cases the number of them is enormous, and the curious flesh-like masses passed hardly require a microscope for due recognition. Ova are not often to be found in the fæces, and in cases in which the symptoms are mainly those of rectal and intestinal irritation, the disease is very difficult to diagnose unless the urine is kept constantly under observation. The same remark applies to cases which are occasionally met with in which the patient is under the belief that he is suffering from malaria, and has in fact attacks of fever due to very slight and inconstant absorption of septic products. As regards the finding of ova in fæces, it may be noted that Manson[3] last year met with a case in private practice, in which ova were found in the fæces and nowhere else. It is not stated in the report that the urine had been examined very frequently, but the patient was not conscious of ever having had hæmaturia. The spines of the ova found were lateral and not apical, a circumstance which has been noted elsewhere when ova has been recovered from the intestinal tube. A further interesting point about Manson's case is that the patient was a well-to-do European resident in the West Indies. This and a case reported by Williamson[4] in a patient from an inland village in Cyprus make it evident that the disease must be borne in mind in dealing with other than patients from Africa.

The prognosis in boys below the age of puberty is good, Guillemard reporting that in Natal they apparently get cured naturally about the time of the onset of puberty; but with adults the case is different. The life of the parasite usually corresponds with that of the patient, and adult patients once affected rarely become quite sound again. The existence of the disease, however, is not always incompatible with a fairly healthy existence, and Guillemard has quoted one which lasted to his knowledge for 18 years. The complications previously referred to, however, are always liable to occur, and the pain and increased hæmaturia caused by active exercise, and especially anything like horse riding, incapacitate the patient from leading an active life.

Since there is no remedy at present known which is capable of either destroying the parasite or effectually dislodging it from the body, the treatment resolves itself into that of symptoms.

Urotropin has been given an extended trial, but does not appear to have had any appreciable effect except as a useful urinary antiseptic in cases complicated with cystitis. The most hopeful drug seems to be methylene blue, the use of which Lieutenant Lelean, R.A.M.C., described before the Medical Society of London last year. The best way to use it is to give three doses of four grains each on one day every week, and on the other six to administer benzoate of ammonia in doses of 10 to 15 grains. The methylene decidedly checks the hæmaturia, and patients experience much relief from its administration, while the benzoate lessens the irritability of the bladder.

[1] Diieases of Warm Climates. [2] Clin. Jour., June 24, 1903. [3] Brit. Med. Jour., Dec. 20, 1902. [4] Ibid., May 3, 1902.

